# Parkinson's Disease and Homocysteine: A Community-Based Study in a Folate and Vitamin B12 Deficient Population

**DOI:** 10.1155/2016/9539836

**Published:** 2016-08-31

**Authors:** Zhang Wei, Wang Tiandong, Li Yang, Meng Huaxing, Min Guowen, Fang Yalan, Niu Xiaoyuan

**Affiliations:** Department of Neurology, First Hospital, Shanxi Medical University, No. 85, Jiefang Nan Street, Yingze District, Taiyuan 030000, China

## Abstract

*Background*. Homocysteine (Hcy) levels were higher in patients with Parkinson's disease (PD). This could be partially explained by levodopa treatment. Whether untreated PD patients have higher Hcy levels is contradictory.* Methods.* A community-based study was conducted using a two-stage approach for subjects ≥ 55 years to find PD patients in 3 towns of Lüliang City. Blood samples were collected. Serum Hcy, folate, and vitamin B12 concentrations were measured. For each untreated PD patient, 5 controls were selected matched with age and sex to evaluate the relationship between Hcy levels and PD.* Results*. Of 6338 eligible residents, 72.7% participated in the study. 31 PD cases were identified. The crude prevalence of PD for people ≥ 55 years was 0.67%. Blood samples were collected from 1845 subjects, including 17 untreated PD patients. There was no difference for concentrations of serum Hcy, folate, and vitamin B12 between cases and controls (*P* > 0.05). In univariate and multivariate analysis, there was significant inverse relation between PD and current smoking (*P* < 0.05). No other factor was significant statistically.* Conclusions*. The prevalence of PD was comparable to earlier studies in China. Hyperhomocysteinemia was not a risk factor of PD, as well as folate and vitamin B12 deficiency.

## 1. Introduction

Parkinson's disease (PD) is the second most common neurodegenerative disease after Alzheimer's disease in the elderly [1]. The estimated number of individuals above age of 50 with PD in the world was between 4.1 and 4.6 million in 2005 [2]. As aging population grows, the number will double to between 8.7 and 9.3 million by 2030 [2]. However, the etiology of PD remains unknown after decades of research. The interracial rates of PD are the same among communities in a common environment [3]. Nevertheless, the prevalence ratios for blacks in different countries varied obviously [4]. Therefore, environmental factors may be more important than genetic factors. Epidemiological studies also indicate that environmental factors, such as cigarettes smoking, are involved in the development of PD [5].

Homocysteine (Hcy) is formed by demethylation of methionine. Hyperhomocysteinemia plays a role in the development of some diseases, such as cardiovascular diseases and neurodegenerative diseases [6–8]. In vitro and in vivo studies indicated that Hcy contribute to the pathogenesis of mesencephalic dopaminergic neuronal death occurring in PD [9]. However, available data from clinical studies were contradictory [9]. Hcy levels were 30% higher in PD patients compared to controls [10]. This could be partially explained by regular treatment of levodopa [11]. Investigating the Hcy levels of naive PD patients may give some clues on the causal relationship between Hcy and PD.

The population living in Lüliang City, Shanxi Province, China, had inadequate folate and vitamin B12 intake [12] which may lead to high prevalence of hyperhomocysteinemia [13]. Meanwhile, because of low Development and Life Index [14], we presumed that most PD patients were undiagnosed and untreated. Thus, it was a proper area to research the relationship between Hcy and PD. In addition, there was no study regarding the prevalence of PD in rural North China. Similar studies were performed in large cities, such as Beijing and Shanghai, and areas around them in China before.

Aim of this study was bifold: (1) to find PD patients and estimate the prevalence of PD in Lüliang City; (2) to examine whether untreated PD patients had higher Hcy levels and the association between hyperhomocysteinemia and PD risk by case-control method.

## 2. Methods

### 2.1. Population Description

The study was performed in Lüliang City, Shanxi Province, China. Lüliang, a mountainous region, is located in the midwest of Shanxi Province which is an underdeveloped area of China. There are approximately 3,720,000 inhabitants, 80% of whom live in rural areas. 148 rural towns belong to Lüliang City, which can be divided into three categories (North, Center, and South) by orientation. Three townships (Kangcheng, Gaojiagou, and Caijiaya) from all three categories were selected as the target populations because of good cooperation of local governments. There was no tap water supply in the 3 towns and people all drank well water there.

### 2.2. Eligibility Criteria

We obtained a census list for 2012 from the household registry department of each town. The study was restricted to residents at age of 55 years or older (birth before 1958-01-01). Residents living in a Lüliang City residence less than 2 months per year were excluded from the study.

### 2.3. General Study Design

The study was performed from August 2012 to December 2012. A free medical consultation in local village health clinics was conducted to improve participation. About 10 days before the survey information on the opportunity to participate was published in local communities via leaflets and posters. Community workers contacted the residents via telephone calls or house visits one or two days before the evaluation to notify them of the time and place of examination. All respondents gave their written informed consent.

A two-stage community-based survey design was employed to detect PD patients. In the first stage, trained interviewers (doctors and senior clinical medical students) administrated a screening questionnaire [15] for PD in local health clinics. Demographic and medical information was also collected, including smoking, tea drinking, and alcohol drinking habits. If targeted residents did not appear in the local health clinics, interviewers visited their house once to administrate the questionnaire. Residents not at home during home visits were considered to have dropped out of the study. The questionnaire contains 9 questions. Residents who responded positively to at least 2 of the 9 questions were selected for the second stage. Sensitivity of the questionnaire was measured in a sample of 47 patients affected by PD; specificity was investigated in 217 outpatients free of parkinsonism. The sensitivity was 97.9% and the specificity was 73.7%. For reducing the false negative rate, subjects who gave positive answers to the question “Do your arms or legs shake?” entered into the second stage as well. We also asked the residents regarding whether levodopa was took and PD was diagnosed before, and subjects giving a positive answer of either question entered into the second stage.

In stage 2, a structured clinical workup comprising the Unified PD Rating Scale [16], a neurologic examination, and standardized history taking, was used to establish the diagnosis of parkinsonism and the classification of parkinsonism. Neurologists from First Hospital, Shanxi Medical University, performed the examinations in local health clinics or at home. Diagnosis of parkinsonism required the presence of bradykinesia and at least one of the following: muscular rigidity, rest tremor, or postural instability [17]. PD was diagnosed according to the United Kingdom Parkinson's Disease Society Brain Bank clinical diagnostic criteria [17]. The staging of PD was assigned according to the Hoehn and Yahr scale [18]. Non-PD parkinsonism including vascular parkinsonism, drug-induced parkinsonism, multiple system atrophy, progressive supranuclear palsy, and other parkinsonism types was diagnosed according to descriptions published previously [19].

Blood samples were collected from subjects who agreed. Approximate 10 mL venous fasting blood samples were obtained from subjects who appeared in the local village health clinics in the early morning hours after a 12 h fast by trained nurses in the field. For PD patients who could not come to the field, the fasting blood samples were collected at home. The serum samples were separated within 30 min of collection by centrifugation (4°C, 20 min, at 2000 RPM) in the field or at home and transported in a cooler to the First Hospital, Shanxi Medical University, Taiyuan, China, where the samples were stored at −70°C until analyzed. Serum total homocysteine (Hcy), folate, and vitamin B12 were measured at the First Hospital, Shanxi Medical University, Taiyuan, China. Hcy was measured by an enzyme cycling method using a Beckman UniCel DxC 800 Synchron Clinical System Analyzer (Beckman Coulter, Inc.). Serum folate and vitamin B12 were measured simultaneously by a radioassay kit (MP Biomedical, Inc.).

Among subjects with blood samples collected, for each untreated PD patient, 5 controls were selected matched with age (±2 years) and sex randomly.

### 2.4. Statistics

For case-control study, current smokers were defined as subjects who smoked during investigation or quitted smoking for less than 3 months. Current alcohol drinkers were defined similarly. Serum Hcy, folate, and vitamin B12 were categorized into binary variable according to the medians.

Prevalence rates were calculated for PD and expressed as percentages. The 95% confidence intervals (CI) were obtained. In addition, prevalence rates standardized to the age composition of the 2010 China census were also calculated. Linear-by-linear association chi-square test was used to test significance of linear relationship between prevalence and age. Differences between categorical variables were calculated using Pearson's chi-square test. Student's *t*-test or Mann-Whitney *U* test was used to compare continuous variables depending on the distribution types. Univariate logistic regression was used to estimate the odds ratios (ORs) and 95% CI for PD. Multivariate logistic regression was used to analyse all the risk factors. A *P* value < 0.05 was considered to be statistically significant. Data were analysed using the Statistical Package for Social Sciences (SPSS) software (version 13.0) and EpiCalc 2000.

## 3. Results 

There were 6338 eligible residents living in the three towns in Lüliang City. Of these, 4605 (72.7%) were screened in stage 1. Age and sex distribution for the target population, participants, and participation rates in this study is presented in Table 1. Of the 4605 who were screened, 367 (7.97%) were suspected to have parkinsonism and entered stage 2. In stage 2, 22 (6.0%) cases could not be traced. Of the 345 (94.0%) subjects clinically evaluated by the neurologists, 37 were diagnosed with parkinsonism. Of the subjects with parkinsonism, 31 (83.8%) had PD, 1 (2.7%) had drug-induced parkinsonism, 1 (2.7%) had progressive supranuclear palsy, and 4 (10.81%) had vascular parkinsonism.

Among 31 PD patients, 30 (96.8%) were newly identified and did not receive treatment of levodopa. The other one had been diagnosed with PD and treated with Madopar for 3 years. 20 patients were males and 11 were females. The mean age at onset of PD was 70.37 ± 10.07 years. The mean duration of disease was 3.31 ± 2.95 years. All 31 patients with PD had two or more cardinal signs of parkinsonism; 24 (77.4%) had three or more signs; 8 (25.8%) had all four. In PD cases, 31 (100%) had bradykinesia, 27 (87.1%) had resting tremor, 26 (83.9%) had rigidity, and 10 (32.3%) had impaired postural reflexes. Regarding the Hoehn and Yahr scale, there were 8 (25.8%) patients in stage I, 13 (41.9%) in stage II, 8 (25.8%) in stage III, 1 (3.2%) in stage IV, and 1 (3.2%) in stage V.

The crude prevalence of PD for people aged 55 years or older was estimated to be 0.67% (95% CI 0.46–0.96). The age-standardized prevalence for people aged 55 years or older was 0.67% after being adjusted to the 2010 China census. The age- and sex-specific prevalence of PD is shown in Table 2. The prevalence of PD increased with advancing age (*P* = 0.00). There was no significant difference for PD prevalence between men and women (*P* = 0.08).

Blood samples were collected from 1845 subjects, including 17 untreated PD patients. Prevalence of hyperhomocysteinemia, low folate levels, and low vitamin B12 levels was high [20]. For each untreated PD patient, 5 controls were selected matched with age (±2 years) and sex. General characteristics of cases and controls are presented in Table 3. There was no difference for concentrations of serum Hcy, folate, and vitamin B12 between cases and controls (*P* = 0.341; *P* = 0.396; *P* = 0.696, resp.). Untreated PD patients had lower prevalence of current smoking (*P* = 0.005). The prevalence of current alcohol drinking, tea drinking, and pesticide exposure was not different significantly between cases and controls (*P* = 0.119; *P* = 0.586; *P* = 0.586, resp.)

In univariate analysis there was significant inverse relation between PD and current smoking (Table 4). Current smokers were found to be less likely to have PD (OR = 0.212; 95% CI 0.067–0.674; *P* = 0.009). High homocysteine levels (*P* = 0.070), low folate levels (*P* = 0.427), low vitamin B12 levels (*P* = 0.427), current alcohol drinking (*P* = 0.999), tea drinking (*P* = 0.999), and pesticide exposure (*P* = 0.999) were found not to have a significant relation with Parkinson's disease in this population. In multivariate analysis, similar results were obtained (Table 4).

## 4. Discussion

In China, the most widely accepted prevalence study on PD was conducted in Beijing, Xian, and Shanghai, which were the largest cities of China [21]. However, regional development differences were obvious in China [14]. In addition, environmental factors were important in the pathogenesis of PD. The present population had different characteristics from those in large cities, such as high prevalence of hyperhomocysteinemia. Investigating the prevalence in this area was necessary.

This study employed a two-stage community-based design to estimate the prevalence rate of PD in a Chinese population, which was popular in epidemiological studies on PD [19, 22, 23]. The differences among those studies were chiefly regarding how to screen PD in stage 1 and we utilized a questionnaire with high sensitivity (97.9%) to perform it. The questionnaire also showed high sensitivity (100%) in community-based study [24]. There was a low chance to miss PD patients in stage 1 in our study. A total of 31 cases were ascertained to have PD, which generated a crude prevalence rate of 0.67% for people aged 55 years or older. The result was comparable to earlier studies conducted in Chinese populations in Kinmen, Taiwan (0.587%, ≥50 years) [25], Ilan county, Taiwan (0.368%, ≥40 years) [19], and Beijing, Xian, and Shanghai, China (1%, ≥55 years) [21]. The prevalence of PD in the present population increased with age, which was in line with the previous studies [19, 25].

Hcy employs multiple neurotoxic mechanisms that have been associated with the pathogenesis of neurodegenerative disorders [9]. Among subjects with blood samples in the present study, most (72.1%) had hyperhomocysteinemia. Several studies regarding hyperhomocysteinemia were conducted in China. Compared to the study of Wang et al. in East China (10.5 *μ*mmol/L, 45–75 years) [13], our study revealed a higher Hcy mean (24.6 *μ*mmol/L) [20]. Hao et al. also showed lower Hcy medians (8.8 *μ*mmol/L for South China and 11.4 *μ*mmol/L for North China) [26] than ours (19.3 *μ*mmol/L) [20]. However, the prevalence of PD in this area was not higher than that on behalf of China (1%, ≥55 years) [21], which indicated that Hcy may be not involved in the pathogenesis of PD.

Several studies found elevated plasma Hcy levels in PD patients treated with levodopa [27–29]. Because of the influence of levodopa, naive PD patients were proper cases for the investigation of the true relationship between Hcy and PD. Some studies had evaluated Hcy levels in untreated PD patients before and the results were contradictory [30, 31]. Small sample sizes (range 15–30) might account for the divergence. A meta-analysis including 6 studies found that there was no significant difference in plasma Hcy levels between untreated patients and healthy controls [32]. In the present study, most PD cases (96.8%) were firstly diagnosed and did not receive treatment, which could be partially interpreted by low development degree of local area. There was no difference for Hcy, folate, and vitamin B12 levels between untreated PD cases and controls in this population. Our results were in accord with the meta-analysis. In univariate and multivariate analysis, hyperhomocysteinemia was not a risk factor of PD, as well as folate and vitamin B12 deficiency. Current smoking was found to be a protective factor for PD, which was widely accepted [33, 34]. The above results indicated that Hcy did not play an important role in the pathogenesis of PD. In cohort studies, intake of folate or vitamin B12 was also not related to the risk of Parkinson's disease [35, 36].

Our study had limitations. There were only 17 untreated PD cases in the case-control study and this could lead to bias. Patients from epidemiological study had common background and were more appropriate for case-control study than cases from hospitals. However, the epidemiological study was costly and hard to expand sample sizes. Nevertheless, our results were generally consistent with the previous studies [32, 35, 36] and credible.

## 5. Conclusions

The prevalence of PD was comparable to earlier studies conducted in Chinese populations and increased with age. There was no difference for Hcy, folate, and vitamin B12 levels between untreated PD cases and controls. Hyperhomocysteinemia was not a risk factor of PD, as well as folate and vitamin B12 deficiency. Hcy did not play an important role in the pathogenesis of PD.

## Figures and Tables

**Table 1:** Age and sex distribution of the targeted population, participants and participation rates.

Age, years	Targeted population, *n*			Participants, *n*			Participation rates, %		
	Men	Women	Total	Men	Women	Total	Men	Women	Total
55–64	1600	1607	3207	1128	1198	2326	70.5	74.5	72.5
65–74	905	949	1854	679	741	1420	75.0	78.1	76.6
75–84	563	559	1122	401	366	767	71.2	65.5	68.4
≥85	75	80	155	42	50	92	56.0	62.5	59.4

Total	3143	3195	6338	2250	2355	4605	71.6	73.7	72.7

**Table 2:** Age-specific and sex-specific prevalence of Parkinson's disease.

Age, years	Men		Women		Total	
	*n*	%	*n*	%	*n*	%
55–64	4	0.35	4	0.33	8	0.34
65–74	3	0.44	5	0.67	8	0.56
75–84	11	2.74	1	0.27	12	1.56
≥85	2	4.76	1	2.00	3	3.26

All ages (≥55)	20	0.89	11	0.47	31	0.67

**Table 3:** General characteristics of cases and controls.

Variables	*n* (%) or medians (25th–75th percentile)		*P* value
	Cases *n* = 17	Control *n* = 85	
Age, years	72.0 (62–80)	72.0 (62–78)	0.989
Sex (%)			
Male	11 (64.7)	55 (64.7)	1
Female	6 (35.3)	30 (35.3)	
Town			
Kangcheng	4 (23.5)	32 (37.6)	0.330
Gaojiagou	5 (29.4)	28 (32.9)	
Caijiaya	8 (47.1)	25 (29.4)	
Serum tHcy, *μ*mmol/L	27.45 (19.70–32.81)	20.71 (14.50–31.50)	0.341
≤22.175	5 (29.4)	46 (54.1)	0.063
>22.175	12 (70.6)	39 (45.9)	
Serum folate, nmol/L	6.92 (4.83–11.13)	7.50 (5.55–13.49)	0.396
≤7.375	10 (58.8)	41 (48.2)	0.425
>7.375	7 (41.2)	44 (51.8)	
Serum vitamin B12, pmol/L	132.45 (95.06–198.84)	149.64 (89.79–195.94)	0.696
≤147.295	10 (58.8)	41 (48.2)	0.425
>147.295	7 (41.2)	44 (51.8)	
Current smoking (%)			
Yes	10 (58.8)	74 (87.1)	0.005
No	7 (41.2)	11 (12.9)	
Current alcohol drinking (%)			
Yes	0 (0)	14 (16.5)	0.119
No	17 (100)	71 (83.5)	
Tea drinking (%)			
Yes	0 (0)	6 (7.1)	0.586
No	17 (100)	79 (92.9)	
Pesticide exposure (%)			
Yes	0 (0)	6 (7.1)	0.586
No	17 (100)	79 (92.9)	

**Table 4:** Univariate and multivariate analysis for the association between PD and possible risk factors.

Factors	Univariate results		Multivariate results	
	OR (95% CI)	*P* value	Adjusted OR (95% CI)	*P* value
Serum tHcy concentrations				
≤22.175	1		1	
>22.175	2.831 (0.917–8.738)	0.070	3.013 (0.785–11.560)	0.108
Serum folate concentrations				
≤7.375	1		1	
>7.375	0.652 (0.227–1.874)	0.427	0.627 (0.172–2.286)	0.480
Serum vitamin B12 concentrations				
≤147.295	1		1	
>147.295	0.652 (0.227–1.874)	0.427	0.312 (0.059–1.642)	0.169
Current smoking				
Yes	0.212 (0.067–0.674)	0.009	0.138 (0.034–0.561)	0.001
No	1		1	
